# Supplementary material for: Insights into unique anatomical structures of the ascidian Halocynthia papillosa obtained by multimodal imaging
Source: Commun Biol. 2026 Apr 22;9:557. doi: 10.1038/s42003-026-10102-5 (PMC13099978; doi:10.1038/s42003-026-10102-5)
Supplement: Supplementary file 1 — Description of Additional Supplementary Files [file 42003_2026_10102_MOESM1_ESM.docx]

**Description of Additional Supplementary Files**

File name: Supplementary data 1
Description: Numerical data of spectral intensity measurements on the red and light side of the body of 12 individuals of Halocynthia papillosa in the contracted and relaxed state. Data of one additional individual (“d47”) of which measurements had failed and which was, therefore, excluded from the analysis is shown.

File name: Supplementary data 2
Description: Spectral intensity maxima on the red and light side of the body of 12 individuals of Halocynthia papillosa in the contracted and relaxed state. This dataset comprises the numerical data for figure 4b and the statistical analysis (repeated measures ANOVA, pairwise T-tests) to compare between red and light sides of the tunic and contracted and relaxed states.

File name: Supplementary Movie 1
Description: The video shows an explosion rendering of the MRI data segmented and animated with VG Studio Max (Version 2022.4, 64-bit). The same MRI data is shown in Figure 1 (c). Grey shows the tunic, green shows the endostyle, dark red shows the tentacle ring with an incision to free the view of the tentacles, yellow shows the dorsal tubercle, brown shows the compromise of digestive and genital tubes, and turquoise shows the pharyngeal basket.

File name: Supplementary Movie 2
Description: The animations show the entire data set with segmentations in green (nerve cord), yellow (dorsal strand plexus), and blue (muscle fibers). Different angles and cropping of the nerve show cavities emerging at the beginning of the dorsal strand plexus. The same HiTT data is shown in Figure 6. The rendering and segmentation were performed with VG Studio Max (Version 2022.4, 64-bit).

File name: Supplementary Movie 3
Description: Animation of an oral tentacle with segmentation of nerve tissue (green) and vascularization (red). The rendering and segmentation were performed with VG Studio Max (Version 2022.4, 64-bit).
