# Supplementary material for: Insights into unique anatomical structures of the ascidian Halocynthia papillosa obtained by multimodal imaging
Source: Commun Biol. 2026 Apr 22;9:557. doi: 10.1038/s42003-026-10102-5 (PMC13099978; doi:10.1038/s42003-026-10102-5)
Supplement: Supplementary file 7 — Reporting Summary [file 42003_2026_10102_MOESM7_ESM.pdf]

Corresponding author(s): Mareike Huhn

Last updated by author(s): Mar 25, 2026

Reporting Summary

Nature Portfolio wishes to improve the reproducibility of the work that we publish. This form provides structure for consistency and transparency in reporting. For further information on Nature Portfolio policies, see our Editorial Policies and the Editorial Policy Checklist.

Statistics

For all statistical analyses, confirm that the following items are present in the figure legend, table legend, main text, or Methods section.

n/a	Confirmed	
☐	☒	The exact sample size (*n*) for each experimental group/condition, given as a discrete number and unit of measurement
☐	☒	A statement on whether measurements were taken from distinct samples or whether the same sample was measured repeatedly
☐	☒	The statistical test(s) used AND whether they are one- or two-sided<br>*Only common tests should be described solely by name; describe more complex techniques in the Methods section.*
☐	☒	A description of all covariates tested
☐	☒	A description of any assumptions or corrections, such as tests of normality and adjustment for multiple comparisons
☐	☒	A full description of the statistical parameters including central tendency (e.g. means) or other basic estimates (e.g. regression coefficient) AND variation (e.g. standard deviation) or associated estimates of uncertainty (e.g. confidence intervals)
☐	☒	For null hypothesis testing, the test statistic (e.g. F, t, r) with confidence intervals, effect sizes, degrees of freedom and P value noted<br>*Give P values as exact values whenever suitable.*
☒	☐	For Bayesian analysis, information on the choice of priors and Markov chain Monte Carlo settings
☒	☐	For hierarchical and complex designs, identification of the appropriate level for tests and full reporting of outcomes
☐	☒	Estimates of effect sizes (e.g. Cohen's *d*, Pearson's *r*), indicating how they were calculated

Our web collection on statistics for biologists contains articles on many of the points above.

Software and code

Policy information about availability of computer code

Data collection	Preprocessing software of MR Imaging: ParaVision 360.1, ImageJ was used for contrast adjustments, measurements of dimensions were obtained with Osirix®, 3D visualization and explosion videos were produced with VGStudio Max.<br>HiTT: reconstruction was done with TOMO-CTF software package and volume stitching was done with NRStitcher<br>LAS X (Leica) was used for image acquisition of confocal and Thunder microscopy.<br>Spectral measurements: Data acquisition was conducted with OceanView Spectroscopy software (version 2.0.19-rcl).
Data analysis	HiTT: Data analysis, segmentation and 3D visualization, was conducted using VG Studio Max (Version 2022.4, 64-bit).<br>Spectral measurements: Data analysis (repeated measures two-factorial ANOVA) in R (version 2024.12.0+467) with the packages car, rstatix, dplyr, tidyr, tidyverse, ggplot2, lsr, plyr and stats.<br>No custom code was used.

For manuscripts utilizing custom algorithms or software that are central to the research but not yet described in published literature, software must be made available to editors and reviewers. We strongly encourage code deposition in a community repository (e.g. GitHub). See the Nature Portfolio guidelines for submitting code & software for further information.

Data

Policy information about availability of data

All manuscripts must include a data availability statement. This statement should provide the following information, where applicable:

- Accession codes, unique identifiers, or web links for publicly available datasets
- A description of any restrictions on data availability
- For clinical datasets or third party data, please ensure that the statement adheres to our policy

The datasets generated during and/or analyzed during the current study are available from the corresponding author upon request.

Research involving human participants, their data, or biological material

Policy information about studies with human participants or human data. See also policy information about sex, gender (identity/presentation), and sexual orientation and race, ethnicity and racism.

Reporting on sex and gender	No research involving human participants, their data, or biological material was conducted.
Reporting on race, ethnicity, or other socially relevant groupings	*Please specify the socially constructed or socially relevant categorization variable(s) used in your manuscript and explain why they were used. Please note that such variables should not be used as proxies for other socially constructed/relevant variables (for example, race or ethnicity should not be used as a proxy for socioeconomic status).* *Provide clear definitions of the relevant terms used, how they were provided (by the participants/respondents, the researchers, or third parties), and the method(s) used to classify people into the different categories (e.g. self-report, census or administrative data, social media data, etc.)* *Please provide details about how you controlled for confounding variables in your analyses.*
Population characteristics	*Describe the covariate-relevant population characteristics of the human research participants (e.g. age, genotypic information, past and current diagnosis and treatment categories). If you filled out the behavioural & social sciences study design questions and have nothing to add here, write "See above."*
Recruitment	*Describe how participants were recruited. Outline any potential self-selection bias or other biases that may be present and how these are likely to impact results.*
Ethics oversight	*Identify the organization(s) that approved the study protocol.*

Note that full information on the approval of the study protocol must also be provided in the manuscript.

Field-specific reporting

Please select the one below that is the best fit for your research. If you are not sure, read the appropriate sections before making your selection.

☒ Life sciences ☐ Behavioural & social sciences ☐ Ecological, evolutionary & environmental sciences

For a reference copy of the document with all sections, see nature.com/documents/nr-reporting-summary-flat.pdf

Life sciences study design

All studies must disclose on these points even when the disclosure is negative.

Sample size	Sample size was determined based on parsimony to not having to sacrifice more animals than necessary for obtaining morphological data. We assumed that the structures investigated are highly conserved and, therefore, would not differ visually between individuals. Therefore, for all imaging procedures only 1-2 replicates (i.e. individuals of Halocynthia papillosa) were dissected. For the experimental part of the study (spectral measurements of the tunic) 12 replicates (individuals of H. papillosa) were used, as for this purpose, animals could be kept alive. Here, the number of replicates was determined based on the number of individuals and processing time available.
Data exclusions	One individual was excluded from the spectral measurement data as the measurements were not successful (a maximum intensity of 12.1 was measured (in comparison to a lowest maximum of 4152.0, see complete data in supplementary table 1). The excluded data is available in supplementary table 1.
Replication	Replication and reproducibility were ensured by applying standardized dissection, imaging, and measurement protocols across the specimens. For the experimental component involving spectral measurements of the tunic, replication was achieved using 12 individuals of H. papillosa. These experiments required live specimens, and the number of replicates was determined by animal availability and processing constraints. Consistent experimental conditions were maintained to ensure reproducibility.
Randomization	No allocation into experimental groups was necessary as bright and dark side of each individual was compared within the individual in the experimental part of the study.
Blinding	Blinding was not possible as no group assignment was conducted in the experiment.

Reporting for specific materials, systems and methods

We require information from authors about some types of materials, experimental systems and methods used in many studies. Here, indicate whether each material, system or method listed is relevant to your study. If you are not sure if a list item applies to your research, read the appropriate section before selecting a response.

Materials & experimental systems

n/a	Involved in the study
☒	☐ Antibodies
☒	☐ Eukaryotic cell lines
☒	☐ Palaeontology and archaeology
☐	☒ Animals and other organisms
☒	☐ Clinical data
☒	☐ Dual use research of concern
☒	☐ Plants

Methods

n/a	Involved in the study
☒	☐ ChIP-seq
☒	☐ Flow cytometry
☒	☐ MRI-based neuroimaging

Animals and other research organisms

Policy information about studies involving animals; ARRIVE guidelines recommended for reporting animal research, and Sex and Gender in Research

Laboratory animals	The study did not involve laboratory animals
Wild animals	Halocynthia papillosa is a common Mediterranean ascidian. All procedures were performed by trained personnel and designed to minimize stress and suffering, with anesthesia used for invasive steps. Specimens were collected by scuba diving near Pula, Croatia, in August 2022 and June 2025, following confirmation that no collection permit was required. Animals were carefully detached and transported to Ruhr University Bochum under cooled, aerated conditions to ensure their viability.
Reporting on sex	The species is hermaphroditic.
Field-collected samples	Ascidians were maintained in a 123 L aquarium at low density with continuous water circulation, cooling to 16 °C, and multi-stage filtration. They were fed Nannochloropsis salina six times daily, with regular partial water changes using artificial seawater. Lighting followed a 12 h light–dark cycle under controlled conditions.
Ethics oversight	No animal experimentation approval was required as H. papillosa is a non-cephalopod invertebrate.

Note that full information on the approval of the study protocol must also be provided in the manuscript.

Plants

Seed stocks	No plants were used in this study
Novel plant genotypes	*Describe the methods by which all novel plant genotypes were produced. This includes those generated by transgenic approaches, gene editing, chemical/radiation-based mutagenesis and hybridization. For transgenic lines, describe the transformation method, the number of independent lines analyzed and the generation upon which experiments were performed. For gene-edited lines, describe the editor used, the endogenous sequence targeted for editing, the targeting guide RNA sequence (if applicable) and how the editor was applied.*
Authentication	*Describe any authentication procedures for each seed stock used or novel genotype generated. Describe any experiments used to assess the effect of a mutation and, where applicable, how potential secondary effects (e.g. second site T-DNA insertions, mosiacism, off-target gene editing) were examined.*
